# Correlation between In Vitro Neutralization Assay and Serological Tests for Protective Antibodies Detection

**DOI:** 10.3390/ijms23179566

**Published:** 2022-08-24

**Authors:** Maria Addolorata Bonifacio, Riccardo Laterza, Angela Vinella, Annalisa Schirinzi, Mariangela Defilippis, Francesca Di Serio, Angelo Ostuni, Antonio Fasanella, Maria Addolorata Mariggiò

**Affiliations:** 1 Department of Biomedical Sciences and Human Oncology, University of Bari Aldo Moro Medical School, 70124 Bari, Italy; 2 Clinical Pathology Unit, University of Bari Aldo Moro, 70124 Bari, Italy; 3 Immunohematology and Transfusion Medicine Service, Azienda Ospedaliero-Universitaria Consorziale Policlinico di Bari, University of Bari “Aldo Moro”, 70124 Bari, Italy; 4 Istituto Istituto Zooprofilattico Sperimentale della Puglia e della Basilicata, 71121 Foggia, Italy

**Keywords:** SARS-CoV-2, antibodies, diagnostics, serological test, surrogate virus neutralization test (sVNT), plaque reduction neutralization test (PRNT), ELISA, epidemiology, humoral immunity, ROC curve

## Abstract

Serological assays are useful in investigating the development of humoral immunity against SARS-CoV-2 in the context of epidemiological studies focusing on the spread of protective immunity. The plaque reduction neutralization test (PRNT) is the gold standard method to assess the titer of protective antibodies in serum samples. However, to provide a result, the PRNT requires several days, skilled operators, and biosafety level 3 laboratories. Therefore, alternative methods are being assessed to establish a relationship between their outcomes and PRNT results. In this work, four different immunoassays (Roche Elecsys^®^ Anti SARS-CoV-2 S, Snibe MAGLUMI^®^ SARS-CoV-2 S-RBD IgG, Snibe MAGLUMI^®^ 2019-nCoV IgG, and EUROIMMUN^®^ SARS-CoV-2 NeutraLISA assays, respectively) have been performed on individuals healed after SARS-CoV-2 infection. The correlation between each assay and the reference method has been explored through linear regression modeling, as well as through the calculation of Pearson’s and Spearman’s coefficients. Furthermore, the ability of serological tests to discriminate samples with high titers of neutralizing antibodies (>160) has been assessed by ROC curve analyses, Cohen’s Kappa coefficient, and positive predictive agreement. The EUROIMMUN^®^ NeutraLISA assay displayed the best correlation with PRNT results (Pearson and Spearman coefficients equal to 0.660 and 0.784, respectively), as well as the ROC curve with the highest accuracy, sensitivity, and specificity (0.857, 0.889, and 0.829, respectively).

## 1. Introduction

Since the declaration of the COVID-19 pandemic status by the World Health Organization (WHO), different serological tests to detect antibodies against SARS-CoV-2 have been marketed worldwide [1,2]. Serological tests commercialized so far are based on different reaction chemistries (i.e., chemiluminescent/electro-chemiluminescent, immunochromatographic, and immunoenzymatic methods) and claim the qualitative or quantitative determination of antibodies against viral antigens [3,4]. The number of performed serological tests increased during the first COVID-19 wave to support the study of virus spread. Later on, serological tests were carried out in an attempt to predict the duration of vaccine-induced immunity to address future vaccination strategies and, ultimately, to estimate the protective humoral immunity within a population [5,6]. In February, the European Centre for Disease Prevention and Control (ECDC) recommended the use of serological tests for population surveys in the context of epidemiological studies [7]. Conversely, the prediction of protective immunity has been discouraged on a single patient scale by the same ECDC. Several studies highlight that the immune response, with humoral immunity tightly bound to cell-mediated response, cannot be reduced only to antibody synthesis [8,9]. Indeed, the immune response is a complex and dynamic process, characterized by inter- and intra-individual variability, of which antibodies represent just one of many faces [10].

Nevertheless, recent seroepidemiological studies have reported the opportunity to analyze serological data to gain insights into immune protection from SARS-CoV-2 infection [11,12]. The titers of antibodies with neutralizing activity (NAb) could be determined by the plaque reduction neutralization test (PRNT_50_), which is considered the reference method [13,14]. However, the PRNT_50_ has several disadvantages, i.e., it requires biosafety level 3 laboratories to handle the virus alive, as well as properly skilled operators. Furthermore, it is expensive and time-consuming [15]. Therefore, in an effort to understand the kinetics and dynamics of protective humoral immunity development, surrogate viral neutralization tests (sVNT) have been marketed based on the competitive incubation of patient’s sera and RBD spike proteins with human angiotensin-converting enzyme 2 (ACE2) receptor [16,17]. As a further step, the relation between sVNT outcomes, in vitro PRNT, and immunological tests is being intensively explored [18,19,20].

The aim of this work consists of the study of humoral immunity in a homogenous set of 83 patients severely infected by SARS-CoV-2 at least 14 days after a negative molecular test. Serum samples were tested by the reference method, i.e., PRNT_50_, as well as by three immunoassays (Roche Elecsys^®^ Anti Spike, Snibe MAGLUMI^®^ S-RBD IgG, and Snibe MAGLUMI^®^ S/N IgG tests), and an ELISA sVNT test (EUROIMMUN^®^ NeutraLISA). The results were compared with the PRNT_50_ in terms of correlation through Pearson’s and Spearman’s coefficients. Moreover, ROC curves were built to assess the performance of the serological assays to predict NAb, comparing the results with those of the PRNT_50_ method. Cohen’s Kappa coefficient and agreement percentage were also calculated to study the concordance of the outcomes.

## 2. Results

The diagnostic performances of three serological immunoassays and an ELISA surrogate viral neutralization test (sVNT) were herein assessed on 83 plasma samples. As a reference, the in vitro neutralization test (PRNT_50_) was performed, leading to the classification of the antibody titers reported in Table 1. The same samples were tested with each of the four immunoassays. However, the three automated tests (Roche Elecsys^®^ Anti Spike, Snibe MAGLUMI^®^ S-RBD IgG, and Snibe MAGLUMI^®^ S/N IgG tests) were not performed on 10 samples due to the lack of required serum volumes.

**Figure 1:**
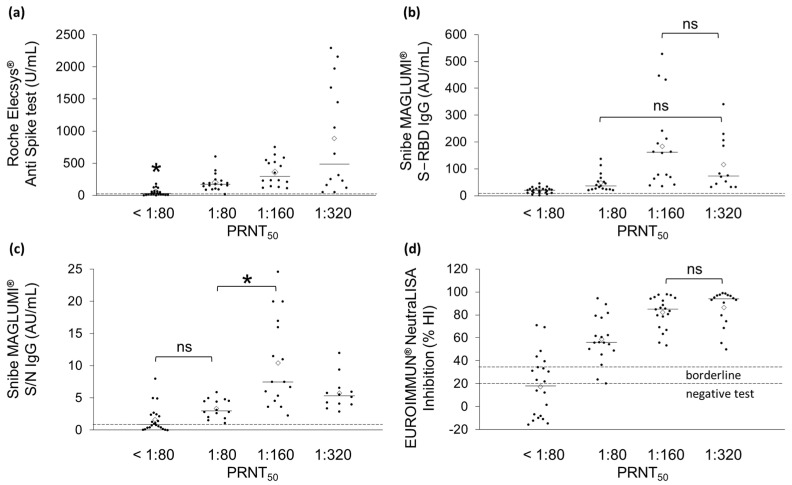
Scatter plots showing the results of the four serological tests, grouped according to PRNT_50_ NAb titers. Solid lines represent median values, white rhombi mean values. (**a**) Roche Elecsys^®^ Anti Spike test (cut off ≥ 0.80 U/mL), (**b**) Snibe MAGLUMI^®^ S-RBD IgG test (cut off ≥ 1.00 AU/mL), (**c**) Snibe MAGLUMI^®^ S/N IgG test (cut off ≥ 1.00 AU/mL), (**d**) EUROIMMUN^®^ NeutraLISA assay (cut off <20% for negative test and up to 35% for borderline values). Dotted lines represent the cut-off for each test. Statistically significant differences between NAb titer groups were assessed by one-way ANOVA and Bonferroni correction (* *p* < 0.05. ns: not significant).

The results obtained by serological methods are summarized in Figure 1, while raw data are available in Table S1 (Supplementary Material). For each assay, the scatter plots (Figure 1) displayed the four groups of NAb titers identified by the PRNT_50_ assay. The Roche Elecsys^®^ Anti Spike test confirmed the presence of anti-SARS-CoV-2 antibodies in all the tested samples. Indeed, no samples falling below the cut-off suggested by the vendor (<0.8 U/mL) were detected (Figure 1a, dotted line). The same applies for the Snibe MAGLUMI^®^ S-RBD IgG test (Figure 1b, dotted line). Conversely, the Snibe MAGLUMI^®^ S/N IgG test classified 12 samples as negative since they fell below the cut-off of 1AU/mL (Figure 1c, dotted line). All the 12 samples belonged to the PRNT_50_ group with the lowest NAb titer (<80). Similarly, 12 samples were labeled as negative (%HI < 20) by the EUROIMMUN^®^ NeutraLISA assay (Figure 1d, dotted line), 11 of which belong to the NAb titer group < 80, and 1 belongs to the NAb titer group > 80. Furthermore, 10 samples provided a borderline result (%HI between 20 and 35%).

The one-way ANOVA with Bonferroni correction highlighted that the Roche Elecsys^®^ Anti Spike test detected significant differences between samples with NAb titers < 1:80 and the three groups of 1:80, 1:160, and 1:320 (Figure 1a, * *p* < 0.05). However, among these three groups, no significant differences were observed. Similarly, the Snibe MAGLUMI^®^ S-RBD IgG test was unable to discriminate between NAb titers > 80 and >320, as well as between NAb titers > 160 and >320 (Figure 1b). Concerning the Snibe MAGLUMI^®^ S/N IgG assay, the groups with NAb titers > 80 and >160 were significantly different (* *p* < 0.05). In addition, the group with an NAb titer < 80 was significantly different from those >160 and >320 (Figure 1c). Finally, the EUROIMMUN^®^ NeutraLISA test was able to distinguish all the groups except those with NAb titers > 160 and >320 (Figure 1d).

**Figure 2:**
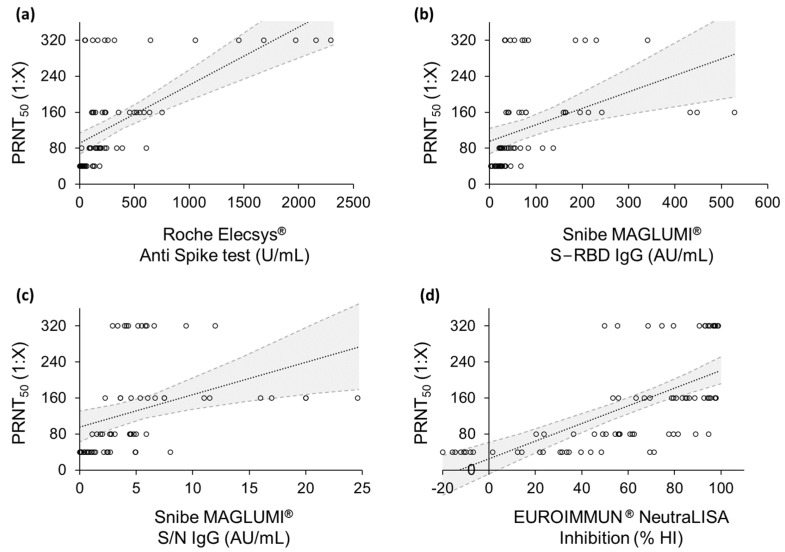
Linear regression analyses related to (**a**) Roche Elecsys^®^ Anti Spike test, (**b**) Snibe MAGLUMI^®^ S-RBD IgG test (**c**) Snibe MAGLUMI^®^ S/N IgG test, (**d**) EUROIMMUN^®^ NeutraLISA assay. Grey areas represent confidence intervals; black dotted lines represent the linear model.

Then, in order to compare the reference method to the outcomes of the serological tests, a linear regression analysis was performed (Figure 2). As also shown in Table 2, the calculated Pearson’s coefficients suggested that the relationship between the results of the PRNT_50_ and those of the EUROIMMUN^®^ NeutraLISA assay, as well as of the Roche Elecsys^®^ Anti Spike test, approached linearity (r = 0.660 and r = 0.617, respectively). Conversely, both the Snibe MAGLUMI^®^ assays displayed a weaker linear correlation with the NAb titers of PRNT_50_ (r = 0.392 and r = 0.364 for SARS-CoV-2 S-RBD IgG and 2019-nCoV IgG, respectively). Nevertheless, calculating the Spearman’s rank correlation coefficients, a positive monotonic trend was confirmed for all the serological assays (Table 3).

**Figure 3:**
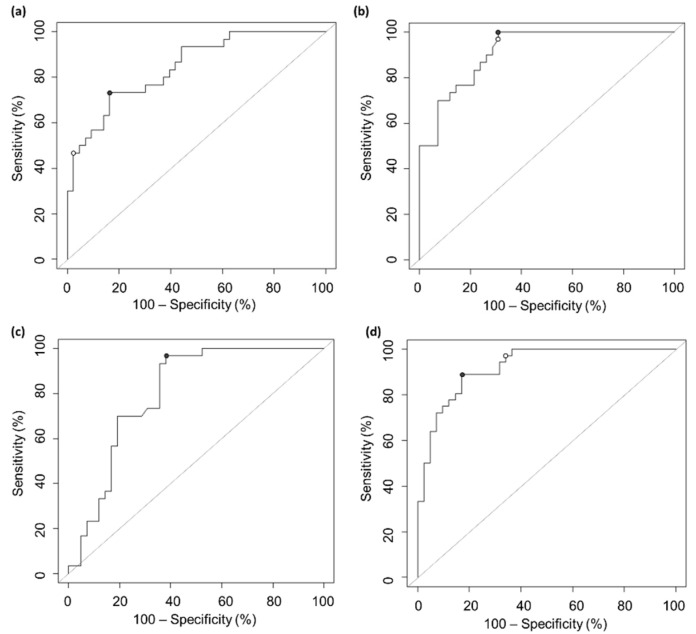
ROC curves relevant to (**a**) Roche Elecsys^®^ Anti Spike test, (**b**) Snibe MAGLUMI^®^ S-RBD IgG test, (**c**) Snibe MAGLUMI^®^ S/N IgG test and (**d**) EUROIMMUN^®^ NeutraLISA test. The black points indicate Youden’s index-optimized cut-off, and the white points indicate the odds-ratio optimized cut-off.

Furthermore, to evaluate the performance of the serological assays to predict NAb titers > 160, ROC curves were built (Figure 3). The cut-off values were chosen by means of Youden’s index (Table 4).

The values of the area under curve (AUC) were >0.8 for each of the four assays. Furthermore, the accuracy was always better than 0.7, as well as sensitivity, while specificity ranged from 0.6 to 0.8 (Table 4). Among the four assays, the EUROIMMUN^®^ NeutraLISA test achieved the best performance, with an AUC of 0.921, indicating good predictive power of the NeutraLISA test. Furthermore, the Youden’s index allowed a maximum accuracy of 0.857, choosing the cut-off % HI equal to 63.3% (Figure 3d, black point). Sensitivity and specificity were both over 0.8 (Table 4). As an alternative, the odds ratio method resulted in a lower cut-off (%HI = 53.5, Figure 3d, white point), maximizing sensitivity in spite of specificity (0.972 and 0.659, respectively), as reported in Table S2. Additionally, the Snibe MAGLUMI^®^ S-RBD IgG test displayed an AUC > 0.9 and reached a sensitivity of 1.000 while its specificity was 0.691 (Table 4). Satisfying results were also obtained for Roche Elecsys^®^ Anti Spike and Snibe MAGLUMI^®^ S/N IgG assays.

Moreover, to explore the concordance between PRNT_50_ and the outcomes of the serological assays, Cohen’s Kappa coefficients were calculated (Table 5). As expected, the EUROIMMUN^®^ NeutraLISA test achieved the highest Kappa coefficient, followed by the Roche Elecsys^®^ Anti Spike assay. Furthermore, the Snibe MAGLUMI^®^ S/N test had a Kappa coefficient equal to 0.913, while the Snibe MAGLUMI^®^ S-RBD IgG reached a value of only 0.650. According to the interpretation provided by McHugh et al. [21], the concordance was almost perfect for all the assays, except for Snibe MAGLUMI^®^ S-RBD IgG, which showed a moderate concordance. These results followed the same trend observed when calculating the positive predictive agreement for each assay (Table 5).

## 3. Discussion

In this work, a detailed statistical analysis was performed to compare the outcomes of four different immunoassays (i.e., three serological tests and an ELISA sVNT) with those of the reference method (PRNT_50_). The ultimate aim of the work is to demonstrate the consistent correlation between an sVNT and the PRNT_50_ assay. Indeed, a simple and reliable serological test could overcome the requirements of PRNT_50_ (i.e., specialized laboratories, several days, skilled operators), opening up new landscapes for large-scale epidemiological studies, even in low-resource settings. In this respect, when choosing a test to assess the humoral immunity against SARS-CoV-2, the ASSURED criteria should be taken into account [22]. Indeed, several methods have been applied to study the interactions between SARS-CoV-2 antigens and neutralizing antibodies, but they are often designed for advanced research facilities. As an example, Sun and co-workers used biolayer interferometry to explore the bond between SARS-CoV-2 Spike protein and human ACE2 receptor in vitro, identifying the key viral residues targeted by neutralizing antibodies [23]. In addition, molecular dynamics simulations proved helpful in the prediction of new spike variants’ behaviors and the effectiveness of human neutralizing antibodies [24]. Furthermore, to develop a high-throughput platform to screen neutralizing antibodies against SARS-CoV-2, Fujimoto et al. carried out two-color fluorescence cross-correlation spectroscopy experiments (FCCS), investigating the quantitative interactions between the spike protein and human soluble ACE2 receptor [25]. Conversely, Rusanen and co-workers labeled the viral spike and nucleoprotein antigens and incubated them with serum samples, developing a “mix and read” immunoassay based on Förster resonance energy transfer (FRET) [26]. However, FRET suffered from lower analytical sensitivity as compared to ELISA tests, so it was unable to detect low antibody responses. For these reasons, several research groups focused on simple immunoassays, especially ELISA-based, trying to establish a correlation between them and the reference viral neutralization test [27,28].

In this work, through the linear regression analysis, a good correlation between the PRNT_50_ and each of the serological assays was observed (Figure 1 and Figure 2). The EUROIMMUN^®^ NeutraLISA test allowed better distinction between the four NAb titer groups identified by the reference method (Table 2 and Table 3). The Spearman’s coefficient confirmed a positive monotone correlation for each studied assay, while the Pearson’s coefficient allowed us to gain insights into the linearity of such a correlation.

Furthermore, considering the FDA recommendation to select hyperimmune plasma with a NAb titer > 160, in this study, the opportunity to discriminate samples above or below this titer was explored through ROC curves (Figure 3). The results, reported in Table 4 and Table 5, indicate the good predictive performance of all the assays thanks to the homogeneity of the examined samples and are in agreement with literature findings [29]. The highest AUC was recorded for the EUROIMMUN^®^ NeutraLISA test, which also provided the best balance of performance in terms of sensitivity and specificity. The cut-offs calculated by the Youden’s index were always higher than those suggested by the vendors, because they aimed at the prediction of NAb titers > 160. Thus, the cut-off selection should be adjusted depending on the assay’s intended use (i.e., seroprevalence studies, rapid selection of candidates eligible as hyperimmune plasma donors).

The goal of predicting NAb titers > 160 was achieved for the patients herein studied, but larger cohort assessments must be performed. The ability to detect different antibody isotypes could be a contributing factor to consider while comparing different assays. In this respect, the results herein obtained are more promising for the EUROIMMUN^®^ NeutraLISA and the Roche Elecsys^®^ Anti Spike tests, which are able to detect all antibody isotypes, while both Snibe MAGLUMI^®^ assays only target IgG molecules.

Furthermore, the emergence of new SARS-CoV-2 variants of concern (VOC) is challenging the knowledge of humoral immunity gained so far. The latest reports underline that the new VOC may elicit an antibody response which could be unmatched by the recombinant RBD molecules present in the immunoassays [30]. Therefore, the detection of NAb targeting non-RBD regions of SARS-CoV-2 spike protein, as well as those targeting non-spike molecules (i.e., the nucleocapsid viral protein), could be useful to assess the protective humoral immunity developed within a population. In this respect, immunoassays such as the Snibe MAGLUMI^®^ S/N IgG assay should provide better results as compared with the Snibe MAGLUMI^®^ S-RBD IgG test. Moreover, functional assays (e.g., sVNT) should be less biased by arising VOC than traditional immunoassays. For the same reasons, hemagglutination tests (HAT) could be easily adjusted to face the issue of VOC [31]. However, HATs are not certified for diagnostics, even if they are considered helpful tools in low-resource settings [32,33].

Considering the updated literature findings and the results herein described, it can be concluded that the EUROIMMUN^®^ NeutraLISA assay, exploring the interaction between the soluble ACE-2 receptor and the patients’ antibodies, could provide functional evidence of NAb presence without requiring a BSL-3 laboratory setting. Indeed, even if the in vitro viral neutralization assay remains the reference method, we demonstrated that the EUROIMMUN^®^ NeutraLISA assay is consistent with the PRNT_50_ assay while having the advantages of being quicker and easier to perform. Indeed, the ELISA setup allows the shortening of testing times while significantly reducing costs as well as the need for specifically skilled operators.

## 4. Materials and Methods

### 4.1. Samples Collection

Serum samples were collected from SARS-CoV-2-infected patients recruited at the Immunohematology and Transfusion Medicine Service. Samples (n = 83, 14 females and 69 males) were selected after a negative molecular test (COBAS^®^ Roche RT-qPCR assay on nasopharyngeal swab) was performed at least 14 days before enrolment. The patients’ median age was equal to 42 years (IQR 31–51). Each sample was immediately processed and stored at −20 °C until tested with the five methods described below, unless otherwise specified (Table S1, Supplementary Material). It cannot be excluded that gender bias might have a relevant impact on the results herein shown.

### 4.2. Serological Tests

#### 4.2.1. Roche Elecsys^®^ Anti Spike Test

According to the manufacturer’s instructions (Roche Diagnostics S.p.A, Monza, Italy), the Roche Elecsys^®^ Anti Spike immunoassay was performed on the COBAS^®^ e411 analyzer (Roche Diagnostics S.p.A, Monza, Italy), building a two-point calibration. The test consisted of an Electro-Chemi-Luminescence Indirect Assay (ECLIA) and included two recombinant RBD antigens, which bound the samples’ antibodies in a double-antigen sandwich setup. Briefly, one recombinant RBD antigen was ruthenylated, while the other one was biotinylated. After 9 min of incubation between the two recombinant antigens and the patient serum, a solid phase was added (i.e., microparticles coated with streptavidin) to ease the magnetic recovery of the double antigen–antibody complexes, taking advantage of the biotin-streptavidin bond. After another 9 min of incubation and a washing step, the complexes reached a measuring chamber, in which a voltage was applied. Thus, electrochemiluminescence was triggered and detected by a photomultiplier. The recorded signal increased directly with the number of antibodies within the sample. The measuring range included values between 0.40 and 250 U/mL, but automated sample dilutions (1:10 and 1:100) extended the upper range of quantification to 25,000 U/mL [34].

#### 4.2.2. Snibe MAGLUMI^®^ SARS-CoV-2 S-RBD IgG Test

The indirect chemiluminescence immunoassay (CLIA) was performed by the MAGLUMI^®^ 800 auto-analyzer according to the manufacturer’s instructions, i.e., the Shenzhen New Industries Biomedical Engineering Co., Ltd. (Snibe, Shenzhen, China) [35]. Briefly, the samples were diluted and incubated under vigorous stirring, with magnetic microspheres coated with the recombinant RBD region of SARS-CoV-2 spike protein. After incubation, the antigen–antibody complexes were attracted by a magnetic field to allow the washing of non-bonded molecules. A second incubation was performed with anti-human IgG antibodies labeled with N-(4-Aminobutyl)-N-ethylisoluminol (ABEI). During incubation, labeled antibodies recognized and bound the samples’ IgG. Magnetic attraction allowed us to precipitate the complexes and perform a second wash-out step. Finally, the starter was provided, triggering a chemiluminescent reaction. A photomultiplier detected the resulting signal as relative light units (RLUs) and the autoanalyzer, after proper calibration, was able to calculate the concentration of IgG within the samples.

#### 4.2.3. Snibe MAGLUMI^®^ 2019-nCoV IgG Test

The Snibe MAGLUMI^®^ S/N IgG test was performed as recommended by Snibe (Shenzhen, China), similarly to the test described above. The only difference was the antigens coating the magnetic microparticles, which belonged to the nucleocapsid and the spike proteins [36].

#### 4.2.4. EUROIMMUN^®^ NeutraLISA Test

The test was manually performed according to the supplier (EUROIMMUN Medizinische Labordiagnostika, Lubeck, Germany). The ELISA assay was carried out in microplate strips, in which the reaction wells were coated with the recombinant SARS-CoV-2 S1/RBD domain, obtained through in vitro culture of HEK-293 cells (Human Embryo Kidney, ATCC Number CRL-1573). The patients’ sera were diluted in the supplied sample buffer, which included a biotinylated, soluble human ACE2 receptor. The latter, being the natural ligand of the RBD spike protein, competed with the NAb of the sample. After 1h of incubation, unbound ACE2 molecules were washed away, and a colorimetric reaction was triggered by the addition of the peroxidase enzyme, labeled with streptavidin. Therefore, the color intensity measured at 450nm was inversely correlated to the concentration of NAb within the sample. Results were expressed as inhibition percentage (%IH), calculated according to Equation (1):

%IH = 100% − (Sample Absorbance × 100%/Blank Absorbance)(1)

For %IH between 20 and 35%, the test result was declared borderline, as recommended by the vendor [37].

### 4.3. Plaque-Reduction Neutralization Test (PRNT50)

The PRNT_50_ was performed at the BioSafety Level 3 laboratories of the Istituto Zooprofilattico Sperimentale as a reference method to assess the patients’ sera neutralization capacity against SARS-CoV-2. According to Wölfel et al. [38], the patients’ sera were pre-treated for 30 min at 56 °C (heat inactivation) and subsequently tested in duplicate. After serial dilutions in OptiPro™ medium (Thermo Fisher Scientific Inc, Milan, Italy), patients’ sera were incubated for 1h at 37 °C with a 100 PFU solution of SARS-CoV-2 (GISAID ID: EPI_ISL 406862). Meanwhile, Vero-E6 cells (African Green Monkey kidney cells, American Type Culture Collection ID: CRL-1587, Manassas, VA, USA) were seeded on 24-well plates at a concentration of 4 × 10^5^ cells/mL and cultured at 37 °C, 5% CO_2_, overnight. The preincubated SARS-CoV-2 viral solutions were added to the Vero-E6-seeded wells, incubating them for 1h at 37 °C. Then, the supernatant was discarded, and a PBS wash was performed. The cultures were supplied with DMEM and 1.2% (*w*/*v*) Avicel^®^ microcrystalline cellulose solution (Sigma Aldrich, Milan, Italy) and left for 3 days at 37°C. After that, the supernatant was discarded, and the cells were fixed in a 6% *v*/*v* formaldehyde solution in PBS for 30 min. Finally, the cell monolayers were washed twice with PBS, stained with a 0.2% *w*/*v* of crystal violet solution, rinsed with PBS, and dried for plaque counting. Neutralizing antibody (NAb) titers were calculated as the corresponding serum dilution eliciting a plaque reduction equal to 50% [39].

### 4.4. Statistical Analyses

Data analysis was performed by R software (version 4.0.1., R Development Core Team), loading the packages ggplot2, ROCR, cutpointr, pROC, and vcd [40,41,42,43,44]. The level of statistical significance was set at *p* < 0.05. The Tukey method was used to identify and remove outliers. One-way ANOVA, followed by Bonferroni correction for multiple comparisons, was performed to study the results of the four serological tests. Linear regression and Spearman coefficient analyses allowed us to correlate the outcomes of the serological methods tested as compared to PRNT_50_. Furthermore, ROC curves were built to assess the ability of a serological test to identify a neutralizing antibody titer ≥ 1:160. The cut-off was calculated using the Youden’s Index and the odds ratio, while Cohen’s Kappa coefficient and positive predictive agreement were calculated to assess the concordance of the obtained results.

## Figures and Tables

**Table 1:** Results of the PRNT_50_ assay, performed on 83 patients, after healing from severe SARS-CoV-2 infection.

Neutralizing Antibody Titers	Number of Samples
<80	25
>80	20
>160	21
>320	17

**Table 2:** Results of the linear regression modeling. r: Pearson’s coefficient; β_0_: slope; β_1_: intercept.

Test	r	R^2^	*p*-Value	β_0_	β_1_
Roche Elecsys^®^ Anti Spike test	0.617	0.381	1 × 10^−8^	0.129	90.735
Snibe MAGLUMI^®^ S-RBD IgG test	0.392	0.153	1 × 10^−3^	0.365	95.386
Snibe MAGLUMI^®^ S/N IgG test	0.364	0.132	3 × 10^−3^	7.192	95.383
EUROIMMUN^®^ NeutraLISA assay	0.660	0.436	9 × 10^−11^	1.959	25.044

**Table 3:** Spearman’s rank correlation coefficients (r) relevant to PRNT_50_ results and each of the studied serological assays.

Test	r	*p*-Value
Roche Elecsys^®^ Anti Spike test	0.726	8 × 10^−13^
Snibe MAGLUMI^®^ S-RBD IgG test	0.730	8 × 10^−13^
Snibe MAGLUMI^®^ S/N IgG test	0.681	6 × 10^−10^
EUROIMMUN^®^ NeutraLISA assay	0.784	2 × 10^−16^

**Table 4:** Optimized parameters from the ROC curves, calculated by the Youden’s index, relevant to each serological assay.

Test	AUC	Cut-off	Accuracy	Sensitivity	Specificity
Roche Elecsys^®^ Anti Spike test	0.843	210 U/mL	0.794	0.733	0.837
Snibe MAGLUMI^®^ S-RBD IgG test	0.916	32.7AU/mL	0.819	1.000	0.691
Snibe MAGLUMI^®^ S/N IgG test	0.802	2.9 AU/mL	0.764	0.967	0.619
EUROIMMUN^®^ NeutraLISA assay	0.921	63.3%	0.857	0.889	0.829

**Table 5:** Analyses of concordance relevant to each serological assay.

Test	Cohen’s Kappa Coefficient (CI)	Positive Predictive Agreement
Roche Elecsys^®^ Anti Spike test	0.972 (0.916–1.000)	96.7%
Snibe MAGLUMI^®^ S-RBD IgG test	0.650 (0.489–0.811)	69.8%
Snibe MAGLUMI^®^ S/N IgG test	0.913 (0.817–1.000)	93.3%
EUROIMMUN^®^ NeutraLISA assay	0.975 (0.925–1.000)	97.4%

## Data Availability

All reported data are herein available. Raw data are available from the corresponding author, on reasonable request.

## Supplementary Materials

The following supporting information can be downloaded at: https://www.mdpi.com/article/10.3390/ijms23179566/s1.
