# Supplementary material for: Correlation between In Vitro Neutralization Assay and Serological Tests for Protective Antibodies Detection
Source: Int J Mol Sci. 2022 Aug 24;23(17):9566. doi: 10.3390/ijms23179566 (PMC9455156; doi:10.3390/ijms23179566)
Supplement: Supplementary file 1 [file ijms-23-09566-s001.zip › ijms-1852881-supplementary.pdf]

Correlation between In Vitro Neutralization Assay and Serological Tests for Protective Antibodies Detection

Maria Addolorata Bonifacio [1,†], Riccardo Laterza [1,†], Angela Vinella [1], Annalisa Schirinzi [2], Mariangela Defilippis [3], Francesca Di Serio [2], Angelo Ostuni [3], Antonio Fasanella [4] and Maria Addolorata Mariggiò [1,*]

[1] Department of Biomedical Sciences and Human Oncology, University of Bari Aldo Moro Medical School, 70124 Bari, Italy;
[2] Clinical Pathology Unit, University of Bari Aldo Moro, 70124 Bari, Italy;
[3] Immunohematology and Transfusion Medicine Service, Azienda Ospedaliero-Universitaria Consorziale Policlinico di Bari, University of Bari "Aldo Moro", 70124 Bari, Italy;
[4] Istituto Istituto Zooprofilattico Sperimentale della Puglia e della Basilicata, 71121 Foggia, Italy;
* Correspondence: mariaaddolorata.mariggio@uniba.it
† These authors equally contributed to the work.

Table S1. Samples tested and quantitative data recorded through each test. ND = not determined.

Patient ID	PRNT (1:X)	Roche Elecsys® Anti Spike test (U/mL)	Snibe MAGLUMI® S-RBD IgG test (AU/mL)	Snibe MAGLUMI® S/N IgG test (AU/mL)	EUROIMMUN® NeutraLISA assay (% IH)
1	320	ND	ND	ND	98,5
2	320	52	32,7	9,41	93,2
3	320	649	206,0	5,50	74,5
4	320	1975	185,5	3,35	79,6
5	320	228	83,0	4,30	97,3
6	320	2158	341,0	12,00	96,6
7	320	257	75,5	5,80	93,3
8	320	ND	ND	ND	98,9
9	320	ND	ND	ND	55,4
10	320	48	33,0	5,94	30,1
11	320	1453	791,7	14,94	97,7
12	320	118	52,9	4,14	95,2
13	320	1056	44,7	3,97	68,5
14	320	164	70,4	5,14	90,7
15	320	317	33,1	2,90	49,9
16	320	1681	230,0	6,60	94,4
17	320	2294	713,0	40,13	97,1
18	160	459	195,0	11,00	94,8
19	160	500	432,0	20,00	92,6
20	160	121	163,2	4,54	53,5
21	160	354	164,0	3,60	85,4
22	160	234	78,0	17,00	86,3
23	160	549	212,7	11,50	95,5
24	160	521	159,6	7,50	97,4
25	160	228	41,3	5,37	55,7
26	160	210	78,5	24,60	80,7
27	160	637	242,2	7,48	97,8
28	160	110	35,9	6,68	88,6
29	160	237	63,1	2,24	79,6

30	160	129	39,0	3,60	63,3
31	160	752	447,0	16,00	94,2
32	160	ND	ND	ND	84,7
33	160	ND	ND	ND	23,6
34	160	ND	ND	ND	66,9
35	160	ND	ND	ND	78,7
36	160	ND	ND	ND	69,4
37	160	588	528,0	20,00	97,6
38	160	143	69,0	6,00	83,4
39	80	338	21,0	4,50	23,6
40	80	179	54,0	2,80	60,8
41	80	104	37,0	2,80	62,5
42	80	93	32,0	4,80	61,8
43	80	20	ND	ND	-37,1
44	80	92	20,3	2,01	56,0
45	80	167	23,4	3,10	20,3
46	80	146	26,2	1,85	48,9
47	80	88	27,2	4,42	36,4
48	80	231	83,1	16,50	79,5
49	80	155	45,2	2,66	50,4
50	80	194	51,2	4,54	55,8
51	80	192	24,0	25,00	77,5
52	80	389	24,0	1,10	45,4
53	80	608	137,0	5,90	94,6
54	80	188	66,0	14,00	81,6
55	80	153	40,0	5,00	54,4
56	80	245	114,0	1,50	89,2
57	80	ND	ND	ND	56,3
58	80	ND	ND	ND	0 -15,2
59	40	66	34,6	1,12	30,7
60	40	248	67,3	2,13	71,0
61	40	35	14,8	2,40	0 -7,9
62	40	120	24,9	2,71	39,6
63	40	66	45,5	4,96	69,3
64	40	31	11,6	0,35	0 -6,8
65	40	11	19,6	0,71	0 -10,8
66	40	183	17,7	2,48	14,1
67	40	11	12,0	0,11	0 -12,2
68	40	138	33,0	1,40	43,8
69	40	11	11,0	4,90	0 -9,9
70	40	6	4,4	0,06	0 -15,8
71	40	9	11,0	9,70	0 -20,0
72	40	316	27,0	0,80	34,5
73	40	10	17,0	8,00	0 -14,6
74	40	14	25,0	1,26	1,5
75	40	48	22,0	0,97	23,3
76	40	26	22,0	0,40	12,4

77	40	17	25,0	0,06	33,5
78	40	45	32,0	13,00	48,5
79	40	1	10,0	0,01	0 -36,2
80	40	54	22,0	34,00	31,5
81	40	17	5,9	0,42	0 -20,3
82	40	4,15	2,1	0,16	0 -29,529
83	40	123	21	0,61	21,826

Table S2. Optimized parameters from the ROC curves, calculated by odds ratio, relevant to each of the serological assay.

Test	AUC	Cut-off	Accuracy	Sensitivity	Specificity
Roche Elecsys® Anti Spike test	0.843	459 U/mL	0.767	0.467	0.977
Snibe MAGLUMI® S-RBD IgG test	0.916	33.0 AU/mL	0.806	0.967	0.691
Snibe MAGLUMI® S/N IgG test	0.802	2.9 AU/mL	0.764	0.967	0.619
EUROIMMUN® NeutraLISA assay	0.921	53.5 %	0.805	0.972	0.659
